# Decoding Digital Discourse Through Multimodal Text and Image Machine Learning Models to Classify Sentiment and Detect Hate Speech in Race- and Lesbian, Gay, Bisexual, Transgender, Queer, Intersex, and Asexual Community–Related Posts on Social Media: Quantitative Study

**DOI:** 10.2196/72822

**Published:** 2025-05-12

**Authors:** Thu T Nguyen, Xiaohe Yue, Heran Mane, Kyle Seelman, Penchala Sai Priya Mullaputi, Elizabeth Dennard, Amrutha S Alibilli, Junaid S Merchant, Shaniece Criss, Yulin Hswen, Quynh C Nguyen

**Affiliations:** 1 Department of Epidemiology and Biostatistics University of Maryland, College Park College Park, MD United States; 2 Department of Computer Science University of Maryland, College Park College Park, MD United States; 3 Department of Health Sciences Furman University Greenville United States; 4 Department of Epidemiology and Biostatistics University of California, San Francisco San Francisco United States

**Keywords:** multimodal machine learning, social media analysis, sentiment analysis, hate speech detection, cultural determinants of health, memes, public health, artificial intelligence, AI

## Abstract

**Background:**

A major challenge in sentiment analysis on social media is the increasing prevalence of image-based content, which integrates text and visuals to convey nuanced messages. Traditional text-based approaches have been widely used to assess public attitudes and beliefs; however, they often fail to fully capture the meaning of multimodal content where cultural, contextual, and visual elements play a significant role.

**Objective:**

This study aims to provide practical guidance for collecting, processing, and analyzing social media data using multimodal machine learning models. Specifically, it focuses on training and fine-tuning models to classify sentiment and detect hate speech.

**Methods:**

Social media data were collected from Facebook and Instagram using CrowdTangle, a public insights tool by Meta, and from X via its academic research application programming interface. The dataset was filtered to include only race-related terms and lesbian, gay, bisexual, transgender, queer, intersex, and asexual community–related posts with image attachments, ensuring focus on multimodal content. Human annotators labeled 13,000 posts into 4 categories: negative sentiment, positive sentiment, hate, or antihate. We evaluated unimodal (Bidirectional Encoder Representations from Transformers for text and Visual Geometry Group 16 for images) and multimodal (Contrastive Language-Image Pretraining [CLIP], Visual Bidirectional Encoder Representations from Transformers [VisualBERTs], and an intermediate fusion) models. To enhance model performance, the synthetic minority oversampling technique was applied to address class imbalances, and latent Dirichlet allocation was used to improve semantic representations.

**Results:**

Our findings highlighted key differences in model performance. Among unimodal models, Bidirectional Encoder Representations from Transformer outperformed Visual Geometry Group 16, achieving higher accuracy and macro–*F*_1_-scores across all tasks. Among multimodal models, CLIP achieved the highest accuracy (0.86) in negative sentiment detection, followed by VisualBERT (0.84). For positive sentiment, VisualBERT outperformed other models with the highest accuracy (0.76). In hate speech detection, the intermediate fusion model demonstrated the highest accuracy (0.91) with a macro–*F*_1_-score of 0.64, ensuring balanced performance. Meanwhile, VisualBERT performed best in antihate classification, achieving an accuracy of 0.78. Applying latent Dirichlet allocation and the synthetic minority oversampling technique improved minority class detection, particularly for antihate content. Overall, the intermediate fusion model provided the most balanced performance across tasks, while CLIP excelled in accuracy-driven classifications. Although VisualBERT performed well in certain areas, it struggled to maintain a precision-recall balance. These results emphasized the effectiveness of multimodal approaches over unimodal models in analyzing social media sentiment.

**Conclusions:**

This study contributes to the growing research on multimodal machine learning by demonstrating how advanced models, data augmentation techniques, and diverse datasets can enhance the analysis of social media content. The findings offer valuable insights for researchers, policy makers, and public health professionals seeking to leverage artificial intelligence for social media monitoring and addressing broader societal challenges.

## Introduction

### Background

The rapid growth of social media in the early 21st century has fundamentally transformed how people receive and share information [1]. This seismic change in information systems has been leveraged across various industries, ranging from politics, finance, health care, and education [2-5], allowing investigators to examine how recent technological advancements affect people’s social interactions and understanding of the world [6-9]. In addition, social media provides outstanding opportunities for collecting far-reaching, information-dense, and vast-scale data [6,8], which can be used to measure real-time public opinions and sentiments on a wide range of topics [9-11]. Most notably, social media–derived sentiment measures overcome numerous limitations of traditional survey approaches in capturing social and cultural trends [12], providing deeper insights into sensitive topics and social determinants of health, such as race, gender, and sexual orientation [13]. The sense of anonymity provided by web-based spaces emboldens people to express views they may not express during in-person interactions [14]. Collecting, cleaning, and analyzing these data often demands expertise in artificial intelligence (AI), machine learning (ML), data science, or computer science, posing challenges for public health researchers with limited technical backgrounds. This paper outlines a comprehensive framework for collecting, preprocessing, and analyzing multimodal social media data (eg, text and images) to derive public sentiment, providing a generalizable approach for broader social science research.

Researchers are increasingly turning to social media data to advance research on the impact of social and cultural exposures on health outcomes [15-19]. Historically, measuring cultural norms at the population level has been difficult; however, technological advancements in ML and AI have made it feasible to train and deploy models that can efficiently extract public sentiment measures from large-scale social media datasets. For instance, public health research using social media has demonstrated its utility in capturing temporal changes and geographic differences in population-level attitudes, beliefs, and norms toward marginalized groups [13]. However, extant research has largely focused on harvesting and analyzing the text of social media posts, and many hate detection and sentiment models were designed to classify text-based content that relied heavily on linguistic features to identify abusive language [20,21]. With recent advances, the social media landscape is rapidly evolving to include novel modalities (eg, images, text, and GIFs).

Visual imagery is a powerful means of communication, capable of conveying social, political, and cultural sentiments [22]. Although most social media platforms use safeguards against hateful content, content moderating algorithms are often less effective in identifying such content in images. There has been a proliferation of harmful content on social media in the form of memes [19,23,24]. The term “meme,” originally coined by evolutionary biologist Dawkins [25] to describe a unit of cultural transmission akin to a gene for expressing and spreading ideas, has now become synonymous with web-based content that combines text and images. Recent studies have sought to bridge the gap in social media public sentiment analysis with multimodal hate detection models that combine text and images for improved accuracy. For instance, Das et al [26] aimed to address the limitations of text-focused approaches by incorporating object detection and sentiment analysis to enhance meme classification. Other studies have leveraged vision and language models (eg, Visual Bidirectional Encoder Representations from Transformers [VisualBERTs] and Universal Image-Text Representation) to highlight the critical role of visual meme characteristics in conveying hateful messages, often surpassing the impact of text alone [27]. A study by Habash et al [28] focused on detecting and categorizing misogynous memes into 4 types: stereotypes, shaming, objectification, and violence. The VisualBERT model achieved a *F*_1_-score of 0.722. While various studies developed multimodal AI models for sentiment classification, many major breakthroughs emerged from data competitions hosted by social media companies.

Facebook’s parent company, Meta Platforms, Inc, launched the Hateful Memes Challenge [29], which provided a dataset of memes with “benign confounders” for the expressed intent of challenging unimodal models and advancing multimodal AI approaches. This challenge highlighted the gap between human annotations and ML model performance, with state-of-the-art models achieving only 64.73% accuracy compared to 84.7% for human ratings. This finding emphasizes the complexity of the ML task and highlights significant detection challenges that require significant improvements. In response, the Hate-CLIPper model by Kumar and Nandakumar [30] improved categorization by capturing the interactions between picture and word embeddings using a feature interaction matrix and Contrastive Language-Image Pretraining (CLIP; Open AI) features that better comprehend subtle contextual clues, such as sarcasm. Wu et al [31] developed the TweetEval, CLIP and enhanced cross-attention, cross-mask mechanisms, which leverages transfer learning and a cross-mask mechanism to enhance the integration of visual and textual features and outperforms conventional ensemble approaches by successfully embedding fine-grained features. Building on this work, the ISSUES model uses a pretrained CLIP vision-language model and textual inversion to enhance the semantic capture of memes [32]. It maps images to pseudoword tokens in the CLIP embedding space, creating a comprehensive multimodal representation. The key components of this model include disentangling image and text features and using a multimodal fusion network to achieve state-of-the-art results in the Hateful Memes Challenge and HarMeme datasets. However, researchers have pointed out that findings from these models may not generalize well to other contexts due to the limitations of the Hateful Memes datasets [33], which do not fully capture the diverse ways in which visual and textual content are represented in other real-world contexts across various social media platforms.

Research into meme dataset creation is relatively scarce. A study by Sharma et al [34] highlighted how the complexity of memes on social media, due to their combination of textual, visual, and audio contents, has been underestimated by researchers. They noted that for certain types of memes, there has been a lack of comprehensive datasets that can be used for training. A study from Kirk et al [33] focused on the collection of hateful and nonhateful memes from Pinterest. They highlighted that “memes in the wild” are more diverse than traditional memes, posing challenges for multimodal models. Key challenges include meme caption extraction and memes with pure texts and plain backgrounds. To address the interpretability gap in multimodal hate detection, Hee et al [35] introduced the Hateful Memes Reasoning Dataset, which includes ground-truth explanations to provide contextual reasons for flagged hateful content in memes. By generating these explanations, Hateful Memes Reasoning Dataset aims to assist content moderators in understanding why a meme is classified as hateful [36]. In addition, Hossain et al [37] focused on hate detection in low-resource languages using the Bengali Hateful Memes dataset, which identifies specific hate targets (eg, communities and individuals) within Bengali memes. The Dual Co-Attention Network leverages both text and images, emphasizing that multimodal datasets are essential for understanding hate directed at specific communities in a culturally nuanced way [38]. Building on these findings, this study aims to address current gaps in the literature to provide guidance for social science and public health researchers. Much of the existing research on hateful meme detection has been conducted using the meme dataset from the Facebook AI competition, such as that by Hee et al [36].

### Objectives

We aimed to create our own dataset with memes collected from social media to contribute to the extension of multimodal datasets in this field. While most studies focus on the detection of all types of hateful memes, our research concentrated specifically on developing multimodal models to detect hateful content targeting racial, gender, and sexual minority groups on social media. These social identities are central social determinants of health and increase exposure to multiple forms of oppression and discrimination. In addition, our study went beyond traditional multimodal hate detection models by systematically evaluating unimodal (Bidirectional Encoder Representations from Transformers [BERT; Google LLC] and Visual Geometry Group 16 [VGG-16]; University of Oxford) and multimodal (CLIP, VisualBERT, and intermediate fusion) approaches, providing a comparative analysis of their strengths and weaknesses. To further enhance classification accuracy, we incorporated the synthetic minority oversampling technique (SMOTE) for minority class balancing and latent Dirichlet allocation (LDA) for topic modeling, which are methodologies that have not been extensively explored in multimodal hate and sentiment analysis. We hoped to facilitate research using social media to measure cultural racism, sexism, heterosexism, and cisgenderism to investigate how the social environment supports, creates, and maintains health inequities. Therefore, this study aimed to provide comprehensive and practical guidance for collecting, cleaning, and processing social media data while highlighting the steps we used in training and fine-tuning multimodal ML models to assess the negative sentiment, positive sentiment, hateful content, and antihateful content. In this study, we described challenges and lessons learned during our process of building these multimodal models to facilitate further development in this area. By addressing these methodological gaps and extending previous research, our study contributes a scalable framework that integrates multimodal ML techniques with public health applications.

## Methods

### Data Acquisition and Processing Pipeline

#### Data Collection

We collected social media data from Instagram (Meta Platforms), Facebook (Meta Platforms), and Twitter (subsequently rebranded X, X Corp). Using the Academic Research application programming interface (API) from Twitter, we queried publicly available, US-based, English-language tweets from 2011 to 2023 until the transition of the company to X on July 23, 2023, which led to the discontinuation of the free data access for researchers [39]. The search queries included keywords related to race and lesbian, gay, bisexual, transgender, queer, intersex, and asexual (LGBTQIA+) community. The retrieved JSON objects contained tweet metadata (eg, tweet text, tweet IDs, conversation IDs, time stamp, and image URLs), user metadata (eg, username, user ID, and follower count), and geolocation data. This resulted in a total of 55,8444,310 tweets downloaded.

In addition, we used CrowdTangle (Meta Platforms, Inc), a public insights tool (discontinued on August 14, 2024), to collect publicly available Facebook and Instagram data [40]. Our dataset included posts from 7 million Facebook pages, groups, and verified profiles and 2 million Instagram accounts, including all verified accounts and accounts with at least 50,000 followers. CrowdTangle also provided the ability to search and download data with image attachments containing text. We acquired a total of 3,073,047 posts from January 1, 2016, to June 13, 2024, containing terms related to race and LGBTQIA+. The returned data included post content, image URLs, image captions, post dates, engagement metrics, and other parameters specified in the queries.

#### Data Processing

**Figure 1:**
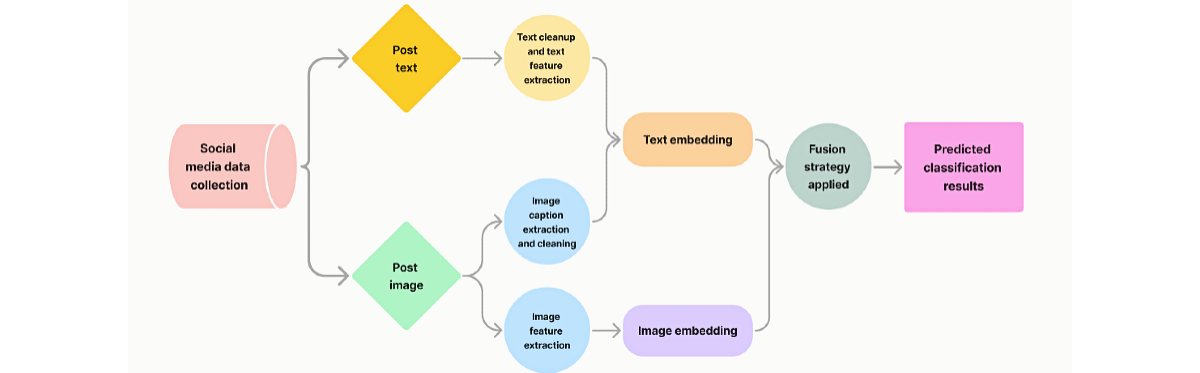
Flowchart of the technical procedure.

The steps needed to collect, process, and analyze these social media data to make the image and text data AI ready are presented in Figure 1. The data acquired from Twitter and CrowdTangle included URLs for posts containing media attachments, such as images, videos, and GIFs. For this study, we filtered the data to include only posts containing images. To download images from CrowdTangle, we used Selenium to initialize a Chrome (Google LLC) browser that navigated to each image URL and extracted the image from the <img> tag. To improve efficiency, we implemented multithreading techniques to download Facebook and Instagram images. For Twitter data, we used the *urllib* library (request function) in Python (Python Software Foundation) to download images. Unlike CrowdTangle data, the downloaded Twitter images were a mix of photos and memes (images with embedded texts). In addition, if download links were available, images containing nudity could be collected as well. We then applied 2 cleaning steps after downloading Twitter data: nudity flagging and meme identification.

Amazon Web Services offers a variety of services relevant to studies that apply ML, such as Amazon Rekognition [41]. Specifically for image and video analysis, Amazon Rekognition allows users to upload and train custom models with minimal memory costs. This integrated system streamlines both model deployment and training on large datasets. Using Amazon Rekognition’s content moderation feature, which can detect inappropriate, unwanted, or offensive content, we filtered out nudity and unsafe content (eg, violence and drugs) across our downloaded images.

In addition, we used the custom label feature to classify memes and regular images, defining memes as images containing text overlays. To achieve this, we manually annotated a dataset with 2988 images labeled “regular images” or “memes,” with which we trained the classification model. This process ensured the removal of generic images of individuals, objects, and landscapes that did not have any textual components. Of the 2988 images, 2393 (80.09%) were used for training, while 595 (19.91%) were reserved for testing. Our meme classification model achieved a macro–*F*_1_-score of 0.96, effectively distinguishing between regular images and memes. We stored filtered memes separately and forwarded them to our annotators for further review. Finally, using *EasyOCR*, an open-source Python library for optical character recognition, we detected and extracted text from the images [42]. It supports >80 languages and provides an easy-to-use API for text extraction.

All ML tasks were performed using the NVIDIA T4 (NVIDIA Corporation) graphics processing unit available on the Google Colab Pro plan. The NVIDIA T4 is a graphics processing unit with advanced capabilities in handling parallel processing tasks. It is commonly used in deep learning studies. The Google Colab Pro plan provided a platform for users to execute high-performance algorithms on their cloud servers instead of using local hardware.

#### Creating Training Data

To create the training dataset for hatefulness and sentiment classification models, we recruited annotators who were aged ≥18 years; proficient in the English language; and active on social media platforms, such as Facebook, Instagram, or X. Annotators were provided with a codebook containing detailed instructions. The first annotation task determined whether a post referenced a specific race or ethnic group, gender identity, or sexual orientation. If none of these identities were referenced, annotators selected “none” and proceeded to the next post. For posts referencing these groups, annotators identified the overall sentiment expressed by the author of the post (neutral, positive, or negative) and evaluated the post’s hatefulness (neutral, hate, or antihate). Before embarking on annotations, we hosted labeling workshops with annotators to review these guidelines and ascertain that the team had a clear understanding of the task and objectives of the project. We defined hateful content as posts that directly or indirectly engender violence, discrimination, hostility, or prejudice against individuals or groups based on their race, ethnicity, gender identity, and sexual orientation. While these types of expressions appear in various ways, including derogatory language, slurs, stereotypes, threats, insults, or demeaning statements, we directed our annotators to look for content that, intentionally or unintentionally, may perpetuate the marginalization or dehumanization of specific groups and threaten their well-being based on their identity.

Conversely, we defined antihate posts as content that opposes or counteracts hate speech, promoting tolerance, inclusivity, or understanding in support of identity groups. These are posts that acknowledge social, cultural, economic, and political structures that further the marginalization of targeted groups based on their identity, aiming to bring awareness and counter these issues. Finally, we defined content where strong hate speech or antihate sentiment was absent as neutral. These posts may include content that is factual or noninflammatory or content that lacks a clear stance on hate speech related to identity groups.

We hosted several sessions with all annotators and collaboratively worked on >400 example posts. These sessions provided the opportunity for open discussions around specific posts until a consensus on how to annotate was achieved. This enabled the team to explore various and pertinent ways in which the different groups of the study were targeted by hateful, antihateful, or neutral content; ask questions; and achieve a shared understanding and framework for evaluating posts relative to targeted identities. While inherent biases might still have influenced the dataset, our approach minimized subjectivity through a structured dispute resolution process and standardized annotation procedures. In addition, we recognized that hate speech detection is an evolving field, and our dataset contributed to ongoing efforts in refining these classification standards.

For the training dataset, each post was assigned to 2 annotators. For posts where consensus was not reached between the 2 annotators, a third annotator coded posts to reach the final codes. To facilitate the annotation process, we used Label Studio Enterprise (HumanSignal) for researchers, an open-source labeling platform for creating and managing labeling projects [43]. In total, we annotated 13,000 social media posts downloaded from Facebook, Instagram, and Twitter for sentiment and hateful content. After removing posts that did not meet our inclusion criteria, we used the remaining 8521 (65.55%) social media posts to train the models.

### Multimodal Architecture

#### Unimodal Models

We evaluated models from 2 categories: single data source ML models (also known as the unimodal models) and multiple modalities of data source models (known as multimodal models). We hypothesized that there would be better model performances from the multimodal model class due to the better use of complementary information and context understanding [44]. For unimodal models, we selected the BERT base model, a powerful pretrained natural language processing model developed by Google, to analyze text data. It is simple to use and can be fine-tuned for various tasks [45]. Compared to more refined models, such as the Robustly Optimized BERT Pretraining Approach or BERTweet, this robust baseline allowed us to evaluate fundamental architecture across varied datasets. Moreover, our decision on BERT was guided by the need for a better, consistent comparison between models, considering our experiments with VisualBERT, which applies text embedding through BERT by default. It allowed us to directly compare the performance of BERT’s text embeddings when used alone in classification problems versus when used as a component and combined with visual data in VisualBERT. For image analysis, we tested VGG-16, which is a convolutional neural network architecture. VGG-16 is widely used in image recognition tasks and can classify 1000 object categories with an accuracy of 92.7% [46].

#### Multimodal Models and Fusion Strategy

For multimodal models, we selected 3 advanced models that incorporated different strategies. The first multimodal model, VisualBERT, is designed to process and integrate both text and image information [47]. VisualBERT is pretrained on the Common Objects in Context dataset, a diverse dataset containing 33,000 images paired with captions, released by Microsoft, and widely used in ML model training [48]. VisualBERT has demonstrated state-of-the-art performance on many vision and language tasks, including Visual Question Answering, a dataset with 265,000 images containing open-ended questions [49]. VisualBERT integrates text embeddings from the BERT model and visual embeddings from the Residual Network (ResNet) with 50 layers by applying an early fusion approach. In this method, multiple modalities are merged before applying the feature extraction process [50]. The embeddings are then concatenated into a single sequence for processing in the transformer layers that use self-attention mechanisms to align and integrate information across modalities. This approach allows the model to dynamically evaluate the importance of different input components, capturing joint representations of text and images. The final classification is accomplished using a classifier layer to predict positive sentiment, negative sentiment, hateful speech, and antihateful speech. To prevent overfitting and enhance the model’s generalization capability, a dropout layer was applied during training.

CLIP was the second multimodal model implemented in this study [51]. Because the model was trained on a wide variety of image and text contents, it performs well on many classification benchmarks without optimization [51]. The CLIP model can process mixed data types as inputs using a contrastive approach to evaluate the relationship between them. It extracts embeddings for both image and text through a shared CLIP encoder and maps them into a common latent space. Unlike VisualBERT, CLIP applies a late fusion method, generating image and text embeddings separately and then projecting them into a shared latent space where they are mapped based on similarities [50]. A fully connected layer combines the output logits with topic distributions for classification tasks.

The third model combines ResNet with 101 layers for image processing and fastText for text processing. We applied an intermediate fusion method where features were concatenated before classification [50]. FastText (Meta AI Research laboratory) generates high-quality text embeddings more efficiently than traditional models [52]. Intermediate fusion allowed us to take advantage of both image and text feature representations simultaneously, often resulting in better model performance compared to using a single combined representation [50]. To support further research in this area, we created GitHub repositories to share code and constructed measures [53].

#### Preprocessing and Image Feature Extraction

For both unimodal and multimodal models’ visual inputs, we resized images to 224×224 pixels using the PyTorch Resize transformation [54]. We then normalized them to the ImageNet standard for red, green, and blue channels (red channel: mean 0.485, SD 0.229; green channel: mean 0.456, SD 0.224; blue channel: mean 0.406, SD 0.225). This normalization reduced variations and aligned with the pretraining conditions of the models.

To address the challenges posed by class imbalance in the training data and to improve generalization to unseen data, we applied augmentation techniques for VisualBERT and CLIP. These techniques generated diverse variations of the minority class image samples [55]. From the Torchvision library in PyTorch, we implemented ColorJitter (PyTorch Foundation) [56], a transformation class that randomly introduces variations, such as brightness, contrast, and saturation, while preserving the core semantics of the images. Other classes from the Torchvision library, such as RandomHorizontalFlip [57], which randomly flips images horizontally, and RandomRotation [58], which rotates images by a random degree within a specified range, were also implemented, further diversifying the minority class representation [59].

For VisualBERT and CLIP, we extracted image features and generated high-dimensional feature embeddings using the ResNet with 50 layers model [60]. ResNet is one of the most commonly used neural networks, and it allows networks to scale to hundreds of layers with competitive accuracy [61]. The features were then processed using adaptive average pooling to aggregate different characteristics and subsequently modified to meet VisualBERT model’s requirements for input dimensions and ensure consistency in feature dimensions [62].

#### Preprocessing and Text Feature Extraction

Each post contained 2 text components: text extracted from the post meme and text from the post content. Both were cleaned by removing nonalphabetical characters, emojis, and stop words and then combined into a single text input before tokenizing. To ensure compatibility with model architectures, we used BertTokenizer for the unimodal model BERT, BERT AutoTokenizer for the multimodal model VisualBERT, CLIPTokenizer for the multimodal model CLIP, and fastText tokenizer for the intermediate fusion model.

#### Model Training and Fine-Tuning

To further mitigate class imbalance for multimodal models, we applied weighted random sampling. This method ensures that each batch in the training phase preserves class balance without data duplication and prevents bias toward the majority class [63].

The acquired training data of 8521 image (memes from posts) and text (meme text and post content) pairs were split into 60% (n=5113) training, 20% (n=1704) validation, and 20% (n=1704) test sets for all the models tested. Cross-entropy [64] loss was applied to the unimodal models (BERT and VGG-16) and the intermediate fusion model. Focal loss [65] calculation was applied to VisualBERT and CLIP to encourage the models to effectively learn from examples that were hard to classify and to more heavily penalize misclassifications of minority classes. Additional optimizations used include AdamW optimizer [54] and gradient calculation techniques. We fine-tuned models using various key hyperparameters, such as epochs (the number of times the models are trained on the full training dataset), batch size (the number of sample data processed before models update their learnable parameters), and learning rate (a parameter that governs how quickly a model learns). The validation set was used to evaluate how well the models were learning at the end of each epoch and help them avoid overfitting. After computing the validation loss for the 3 multimodal models across both negative and positive sentiment as well as hateful and antihateful sentiments, the models achieving the lowest validation loss for the respective classifications were saved and applied to the test set for a final evaluation of models’ performances, using a set of metrics, including accuracy, precision, recall, and macro–*F*_1_-score.

#### Additional Sampling Methods and Feature Testing

To enhance model performance, we incorporated SMOTE [66] and LDA-derived topic distributions [67] into our classification pipeline. These techniques were applied to all multimodal models—CLIP, VisualBERT, and the intermediate fusion model. The integration of SMOTE and LDA was designed to increase the representation of underrepresented classes while enriching the input features with semantic context derived from topic modeling.

Due to the substantial class imbalance in our dataset, particularly in hate and antihate classifications, we applied SMOTE to oversample the minority class. SMOTE generates synthetic samples by interpolating between existing samples in feature space, ensuring that the newly created data remain representative of the minority class. Other text augmentation techniques were considered; however, SMOTE fit better with our purpose. For example, synonym replacement was also commonly used for text augmentation. It substituted words in a sentence with their synonyms to create new variants of existing sentences without changing the original meaning. However, the use of synonym replacement might cause oversimplification because it does not generate any new sentence structure [68]. Simpler techniques, such as data cropping, flipping, and rotation, were tried but did not resolve the problem. SMOTE allowed us to expand the size of the underrepresented class without introducing duplicates, which was critical for preventing overfitting. SMOTE was applied to the training set to balance the class distributions, while the validation and test sets remained unaltered to ensure unbiased evaluation.

To complement multimodal embeddings, we integrated semantic features using LDA. This probabilistic topic modeling technique uncovered latent thematic structures by representing each document as a mixture of topics. The topic model was trained exclusively on the training set text data to avoid data leakage into the validation and test sets. The document-topic distributions generated by LDA served as additional input features, providing probabilistic representations of the text’s thematic content. These topic distributions were concatenated with the multimodal embeddings from each model. Specifically, for the intermediate fusion model, topic features were combined with ResNet with 101 layer image embeddings and fastText text embeddings, enriching the input representation with high-level semantic context. In CLIP and VisualBERT, topic features were integrated with their respective architectures.

#### Quality Control Assessments

In this study, we used accuracy, macro–*F*_1_-score, precision, and recall as model evaluation metrics to assess model performance and facilitate numerical indicators for model comparisons. These evaluation metrics provided an evaluation that aligned with the classification objectives. Precision measured the model’s ability to avoid misclassifying nonhateful content as hateful, which was important for preventing overmoderation. Recall measured the model’s effectiveness in identifying actual instances of hate speech, addressing the primary goal of reducing harmful content. Macro–*F*_1_-score balanced these competing concerns. Macro–*F*_1_-score, precision, and recall were calculated independently for each class and then averaged, ensuring equal weight for all classes regardless of class size [69]. They were especially helpful when data were imbalanced.

### Ethical Considerations

This study was determined not to be a human participant research by the University of Maryland College Park Institutional Review Board (2072551-1). In addition, the social media posts were anonymized, upholding user privacy.

## Results

### Description of Unimodal and Multimodal Models

We evaluated loss functions (a mathematical function used to evaluate deviation between a model’s prediction and the ground-truth labels) and optimizers (algorithms that minimize loss) before selecting hyperparameters that led to the selection of best-performing models, as shown in Table 1.

**Table 1:** Fine-tuned hyperparameters for unimodal and multimodal models.

Model			Epochs, n		Batch size, n		Learning rate		Optimizer		Loss
**Unimodal models**											
	Text: BERT^a^	12		32		0.0001		AdamW		Cross entropy	
	Image: VGG-16^b^	12		32		0.0001		Adam		Cross entropy	
**Multimodal** **m** **odels**											
	Intermediate fusion	6		64		0.0001		AdamW		Cross entropy	
	VisualBERT^c^	15		64		0.00001		AdamW		Focal loss	
	CLIP^d^	15		64		0.00001		AdamW		Focal loss	
**Multimodal models+SMOTE^e^ and LDA^f^**											
	Intermediate fusion	6		32		0.0001		AdamW		Cross entropy	
	VisualBERT	6		32		0.0001		AdamW		Focal loss	
	CLIP	6		32		0.0001		AdamW		Focal loss	

^a^BERT: Bidirectional Encoder Representations from Transformer.

^b^VGG-16: Visual Geometry Group 16.

^c^VisualBERT: Visual Bidirectional Encoder Representations from Transformer.

^d^CLIP: Contrastive Language-Image Pretraining.

^e^SMOTE: synthetic minority oversampling technique.

^f^LDA: latent Dirichlet allocation.

### Performance of Unimodal and Multimodal Models

Table 2 presents the performance metrics of the unimodal models, followed by multimodal models. Within each category of models, results are displayed for specific classification tasks (negative and positive sentiment and hate and antihateful content). There were 2 sets of multimodal models presented, 1 set without LDA and SMOTE and 1 set with LDA and SMOTE.

**Table 2:** Model performance for unimodal and multimodal models.

Model and class				Accuracy		*F*_1_-score (macroaveraged)		Precision (macroaveraged)		Recall (macroaveraged)
**Unimodal** **m** **odels**										
	**Text: BERT^a^**									
		Negative sentiment	0.85		0.82		0.85		0.80	
		Positive sentiment	0.77		0.77		0.77		0.76	
		Hateful	0.91		0.65		0.79		0.6	
		Antihate	0.79		0.64		0.75		0.62	
	**Image: VGG-16^b^**									
		Negative sentiment	0.75		0.69		0.70		0.68	
		Positive sentiment	0.64		0.63		0.63		0.63	
		Hateful	0.82		0.61		0.59		0.64	
		Antihate	0.73		0.59		0.62		0.58	
**Multimodal** **m** **odels**										
	**Intermediate fusion**									
		Negative sentiment	0.76		0.72		0.72		0.71	
		Positive sentiment	0.66		0.63		0.67		0.63	
		Hateful	0.91		0.64		0.76		0.61	
		Antihate	0.75		0.58		0.63		0.58	
	**VisualBERT^c^**									
		Negative sentiment	0.84		0.80		0.83		0.78	
		*Positive sentiment^d^*	*0.76*		*0.76*		*0.760*		*0.76*	
		*Hateful*	*0.91*		*0.62*		*0.80*		*0.59*	
		*Antihate*	*0.78*		*0.61*		*0.74*		*0.60*	
	**CLIP^e^**									
		*Negative sentiment*	*0.86*		*0.83*		*0.84*		*0.82*	
		Positive sentiment	0.74		0.71		0.77		0.71	
		Hateful	0.90		0.59		0.74		0.56	
		Antihate	0.77		0.57		0.70		0.57	
**Multimodal models+LDA^f^ and SMOTE^g^**										
	**Intermediate fusion**									
		Negative sentiment	0.82		0.82		0.91		0.75	
		Positive sentiment	0.71		0.65		0.68		0.63	
		Hateful	0.84		0.79		0.82		0.77	
		Antihate	0.71		0.63		0.57		0.68	
	**VisualBERT**									
		Negative sentiment	0.82		0.44		0.61		0.31	
		Positive sentiment	0.85		0.42		0.57		0.34	
		Hateful	0.84		0.75		0.72		0.77	
		Antihate	0.73		0.42		0.33		0.59	
	**CLIP**									
		*Negative sentiment*	*0.93*		*0.75*		*0.89*		*0.62*	
		*Positive sentiment*	*0.86*		*0.67*		*0.73*		*0.65*	
		*Hateful*	*0.96*		*0.73*		*0.81*		*0.67*	
		*Antihate*	*0.77*		*0.68*		*0.77*		*0.61*	

^a^BERT: Bidirectional Encoder Representations from Transformer.

^b^VGG-16: Visual Geometry Group-16.

^c^VisualBERT: Visual Bidirectional Encoder Representations from Transformer.

^d^Values in italics indicate the best-performing models within these sets.

^e^CLIP: Contrastive Language-Image Pretraining.

^f^SMOTE: synthetic minority oversampling technique.

^g^LDA: latent Dirichlet allocation.

### Comparison of Performance Across Models

For unimodal models, the text-based BERT model outperformed the image-based VGG-16 across various tasks. When predicting negative sentiment, the BERT model achieved a higher accuracy of 0.85 and a macro–*F*_1_-score of 0.82 (Table 2). Similarly, when predicting positive sentiment, the BERT model showed a superior performance with an accuracy and a macro–*F*_1_-score of 0.77. For hateful content classification, the BERT model demonstrated an accuracy of 0.91, with a macro–*F*_1_-score of 0.65. For antihate prediction, the BERT model achieved a higher accuracy of 0.79 with a macro–*F*_1_-score of 0.64 compared to the VGG-16 model’s accuracy of 0.73 and macro–*F*_1_-score of 0.59.

For multimodal models without LDA or SMOTE, when predicting negative sentiment, the CLIP model achieved the best performance with an accuracy of 0.86 and a macro–*F*_1_-score of 0.83. VisualBERT closely followed, with an accuracy of 0.84 and a macro–*F*_1_-score of 0.8 (Table 2). However, the intermediate fusion model performed the worst in this task, with an accuracy of 0.75 and a macro–*F*_1_-score of 0.69. In predicting positive sentiment, VisualBERT outperformed other models with both an accuracy and a macro–*F*_1_-score of 0.76. CLIP achieved an accuracy of 0.74 and a macro–*F*_1_-score of 0.71. The intermediate fusion model had the lowest accuracy of 0.66 and a macro–*F*_1_-score of 0.63. For hate classification, the intermediate fusion model had the best performance with an accuracy of 0.91 and a macro–*F*_1_-score of 0.64. VisualBERT achieved an accuracy of 0.91 and a macro–*F*_1_-score of 0.62. CLIP achieved an accuracy of 0.90 and a macro–*F*_1_-score of 0.59. For antihate classification, VisualBERT performed better, with an accuracy of 0.78 and a macro–*F*_1_-score of 0.61, while CLIP had an accuracy of 0.77 and a macro–*F*_1_-score of 0.57, and the intermediate fusion model had an accuracy of 0.75 and a macro–*F*_1_-score of 0.58.

The implementation of LDA and SMOTE techniques altered model performance patterns. For multimodal models with LDA or SMOTE, when predicting negative sentiment, CLIP showed impressive performance with an accuracy of 0.93 and a macro–*F*_1_-score of 0.75. The intermediate fusion model achieved both an accuracy and a macro–*F*_1_-score of 0.82. VisualBERT showed mixed results, with a strong accuracy of 0.82 but a significantly lower macro–*F*_1_-score of 0.44. When predicting positive sentiment, CLIP again demonstrated the best performance with an accuracy of 0.86 and a macro–*F*_1-_score of 0.67. VisualBERT achieved a higher accuracy of 0.85 but a much lower macro–*F*_1_-score of 0.42 compared to other models, indicating challenges in maintaining balanced precision and recall. Such a pattern suggested that while data augmentation techniques generally improved model performance, their impact varied considerably across different architectures and tasks. For hate classification, CLIP showed marked improvement, achieving the highest accuracy of 0.96 and a macro–*F*_1_-score of 0.73. The intermediate fusion model demonstrated more consistent performance across metrics with the data augmentation techniques, along with an accuracy of 0.84 and a macro–*F*_1_-score of 0.79. For antihate classification, CLIP achieved a better performance with an accuracy of 0.77 and a macro–*F*_1_-score of 0.68. VisualBERT achieved an accuracy of 0.73 and a macro–*F*_1_-score of 0.42, while the intermediate fusion model had an accuracy of 0.71 and a macro–*F*_1_-score of 0.63.

These findings indicated that CLIP’s architecture was particularly robust when enhanced with data augmentation, especially for hate speech and negative sentiment detection. The intermediate fusion model offered the most consistent performance across metrics when using data augmentation, making it potentially more reliable for balanced classification tasks. VisualBERT, while performing well in baseline implementations, may require different optimization strategies when used with data augmentation techniques.

**Figure 2:**
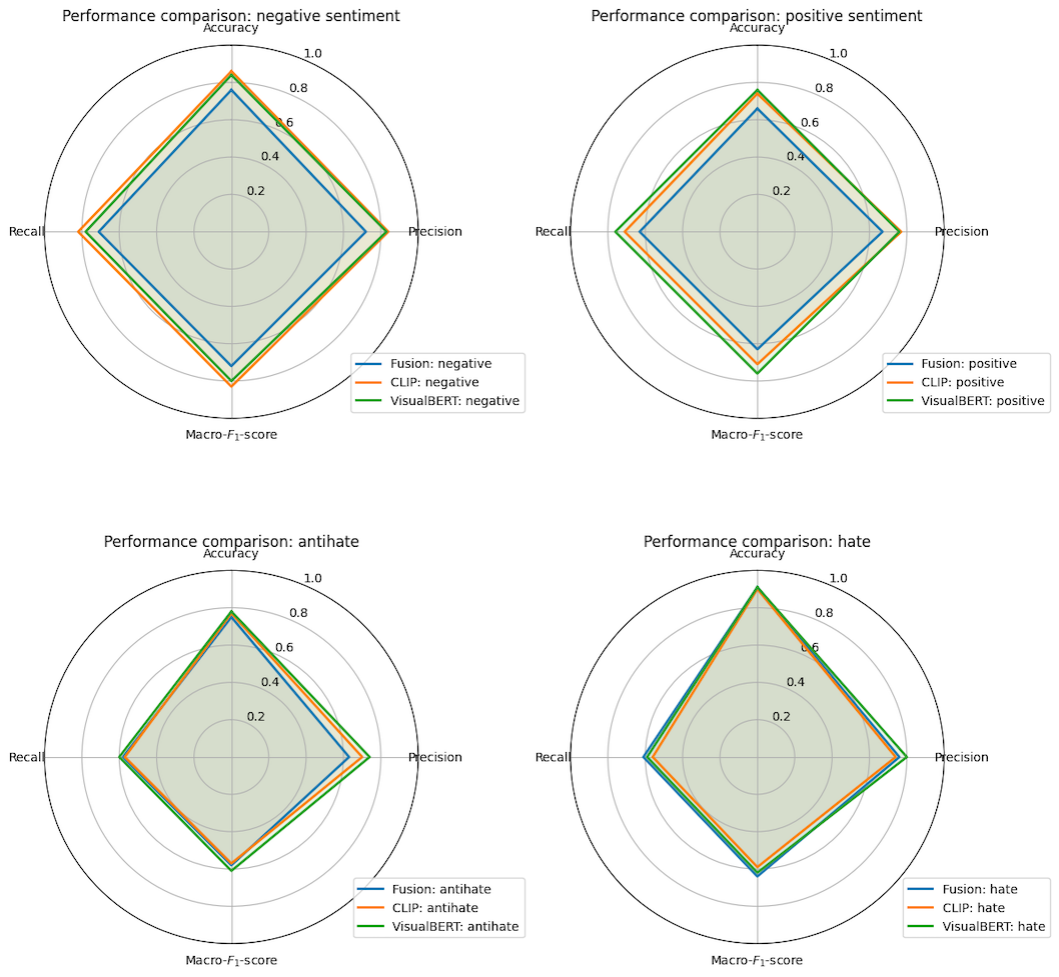
Radar plots of performance metrics for multimodal models. CLIP: Contrastive Language-Image Pretraining; VisualBERT: Visual Bidirectional Encoder Representations from Transformer.

**Figure 3:**
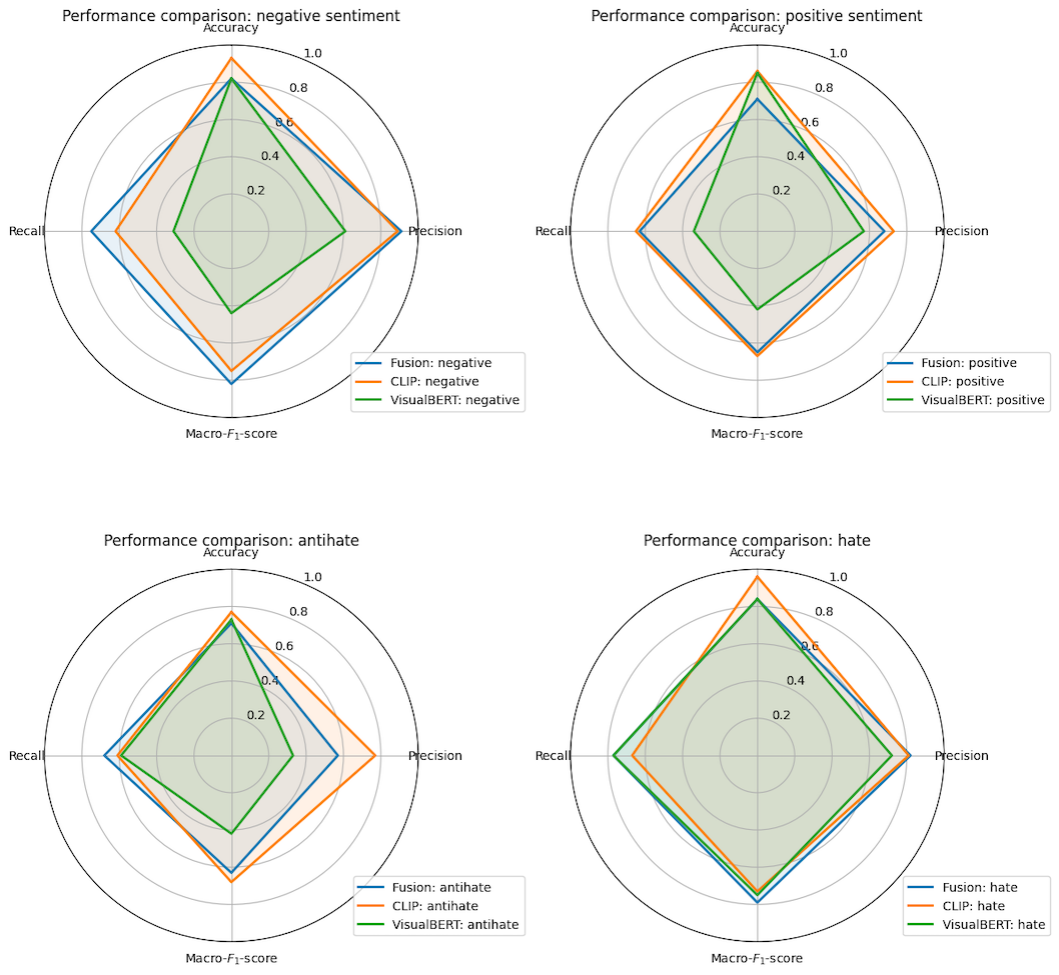
Radar plots of performance metrics for multimodal models with latent Dirichlet allocation and synthetic minority oversampling technique. CLIP: Contrastive Language-Image Pretraining; VisualBERT: Visual Bidirectional Encoder Representations from Transformer.

The radar plots (Figures 2 and 3) provide a comprehensive visualization of the performance metrics, namely accuracy, macro–*F*_1_-score, macro precision, and macro recall, for the 3 models across the 4 categories, namely hate, antihate, positive, and negative. Figure 2 (radar plot) shows that VisualBERT and CLIP were across the board better than the fusion model in classifying sentiment, while the 3 models performed more similarly for hate and antihate comments. Figure 3 displays the accuracy, precision, recall, and macro–*F*_1_-scores for the 3 models augmented with SMOTE and topic distributions. The augmentations tended to decrease performance in the VisualBERT model but increased the performance of CLIP and the fusion models in many categories.

## Discussion

### Principal Findings

The core components of multimodal models are text and image embeddings, which are designed to transform textual and visual data into numerical representations that computers can read and manipulate. These numerical representations capture rich features from the input data, leading to better performance in ML tasks with their capabilities to show semantic meaning between words or pixels. In our multimodal models, text and image embeddings were integrated at certain stages (depending on the fusion strategy), allowing models to leverage multiple data sources for a deeper comprehension of nuanced relationships within data. On the other hand, the experiments on the 2 unimodal models, BERT and VGG-16, helped with analyzing text and image embedding separately. These 2 unimodal models took text-only or image-only data as input and used their built-in embedding features. The comparisons in model performance between multimodal models and unimodal models highlighted the advantages of multimodal embeddings.

The fusion strategy applied in multimodal models also played a critical role in effectively processing data. It decides how information from different data modalities is combined during the modeling process to make a final decision. Many pretrained models have their own default fusion methods, such as VisualBERT using early fusion and CLIP using late fusion. The choice of fusion strategies often significantly impacts the multimodal model performance; therefore, the optimal one can vary and depend on the task needs and data characteristics. This study used the original fusion design for VisualBERT and CLIP.

Findings from this analysis expose distinct patterns in the performance of unimodal and multimodal models, including CLIP, VisualBERT, and intermediate fusion, across sentiment (positive and negative), hate speech, and antihate speech classification. For negative sentiment, CLIP demonstrated superior performance, achieving the highest accuracy (0.86) and macro–*F*_1_-score (0.83), while VisualBERT achieved the highest accuracy and macro–*F*_1_-score (0.76) for positive sentiment, with the intermediate fusion model yielding the lowest accuracy (0.66) and macro–*F*_1_-score (0.63). For hate speech, baseline models achieved similar accuracy levels (0.90), with the intermediate fusion model obtaining the highest macro–*F*_1_-score (0.64), indicating a better balance between precision (0.762) and recall (0.61). For antihate, VisualBERT produced a higher accuracy (0.78) and macro–*F*_1_-score (0.61), demonstrating stronger precision (0.74) compared to all other models. However, the implementation of LDA and SMOTE techniques altered model performance patterns. The intermediate fusion model demonstrated more consistent performance across metrics with data augmentation techniques, notably in hate speech detection (accuracy: 0.84; macro–*F*_1_-score: 0.79) and negative sentiment classification (accuracy: 0.82; macro–*F*_1_-score: 0.82). In contrast, VisualBERT's performance with LDA and SMOTE showed mixed results, with strong accuracy but significantly lower macro–*F*_1_-score values in sentiment classification tasks. For positive and negative sentiments, VisualBERT produced average accuracy values and low macro–*F*_1_-score values, indicating challenges in maintaining balanced precision and recall. This distinct pattern in sentiment classification suggests that data augmentation techniques can improve model performance and vary considerably across classification tasks. Overall, these findings indicate that CLIP is robust when enhanced with data augmentation techniques, especially for hate speech and negative sentiment detection. Furthermore, the intermediate fusion model offers the most consistent performance across all metrics when implementing data augmentation, enhancing its reliability for balanced classification tasks. Finally, while VisualBERT performed well in baseline implementations, this model may require different optimization strategies when coupled with data augmentation techniques. Our findings align with existing research highlighting the strengths of multimodal ML models in analyzing nuanced aspects of complex social media content, such as sentiment, hate, and antihate speech. A previous study by Kumar and Nandakumar [30] introduced the Hate-CLIPper architecture, which effectively combined textual and visual features to enhance classification accuracy, achieving a 0.90 micro–*F*_1_-score on their test set. Similarly, Wu et al [31] explored the capabilities of VisualBERT with the Facebook Hateful Meme dataset and achieved an accuracy of 0.684. The study by Cao et al [70] on the same topic, using the HarMeme dataset [71], achieved an accuracy of 0.75 for VisualBERT and 0.77 for CLIP. Consistent with these insights and evaluations of our results against the existing benchmarks, the robust performance of CLIP and VisualBERT in our study underscores their ability to integrate complementary modalities, promoting nuanced representation and analysis of multimodal data. In addition, the methodological enhancements in the use of data augmentation techniques, such as SMOTE and LDA, can inspire other researchers to further explore new possibilities for advancing this critical area. The findings of this study lay the groundwork for advancing multimodal ML by demonstrating how fusion strategies, diverse datasets, and augmentation techniques shape model performance. The strong results from CLIP and VisualBERT in combining text and images suggest that future research could refine fusion mechanisms to improve adaptability and robustness across diverse social media platforms. Given the complexities of moderating unstructured and adversarial web-based content, future advancements should focus on improving model resilience. This study reaffirms the potential of advanced vision-language models in addressing critical social challenges, including hate speech detection and sentiment analysis.

While social media data can be a powerful lens into society, it is a snapshot that may not be fully representative of the wider population. The data collection includes only publicly available discourse and may not include users with limited web-based presence. Moreover, different platforms have varying user bases. For example, Facebook users skew toward women, whereas X (Twitter) skews toward men [72]. Hence, to account for demographic variance across platforms, we sourced data from 3 platforms rather than 1; however, future studies could further expand their data sources. Consistent with previous literature, our findings highlight the importance of diverse datasets for accurately analyzing harmful memes that reflect complex cultural and social contexts. While previous studies, such as the study by Sharma et al [34], have noted the limitations of existing datasets, our results demonstrate the value of a novel dataset that includes memes targeting minority groups. This inclusivity has provided new insights into the performance of multimodal models (eg, CLIP and VisualBERT) in handling diverse, nuanced social media content.

Building on the work of Chen and Pan [73], which highlighted the challenges of detecting nuanced and imbalanced data, we applied advanced data augmentation techniques (eg, SMOTE and LDA) to effectively address class imbalances. These methods significantly enhanced model performance for minority classes, such as antihate content. However, although VisualBERT was widely recognized as one of the most effective models for vision-language tasks [47], the model exhibited limitations when paired with advanced data augmentation techniques. While the methods improved recall for minority classes, they concurrently diminished precision, revealing trade-offs that merit further investigation. These findings challenge assumptions regarding VisualBERT’s performance and suggest that although augmentation strategies may be beneficial in some contexts, the models may require further refinement to optimize overall performance.

Overall, this study contributes to the growing body of work on multimodal ML by demonstrating the potential of intermediate fusion models, advanced augmentation techniques, and inclusive datasets to enhance the analysis of social media content. By validating existing approaches and challenging conventional assumptions, our findings pave the way for new directions in addressing complex social challenges through multimodal research.

This research offers notable contributions to the field of multimodal sentiment and hate classification. It introduces a robust and scalable pipeline for collecting, preprocessing, and analyzing multimodal social media data, addressing critical challenges in integrating text and image modalities. A notable contribution is the creation of a novel, annotated dataset focusing on content targeting racial, gender, and sexual minority groups. This dataset fills an essential gap in existing resources and expands the scope of social media–based public health research. The study evaluates state-of-the-art models, such as VisualBERT and CLIP, alongside unimodal baselines, such as BERT and VGG-16, providing a comprehensive assessment of architecture-specific strengths and weaknesses. The innovative application of data augmentation techniques, such as LDA for semantic enrichment and SMOTE for class imbalance mitigation, demonstrates the untapped potential of these methods to enhance performance, particularly for underrepresented topics (eg, antihate content). Through rigorous evaluation using metrics (eg, macro–*F*_1_-score, precision, and recall) and transparent dissemination of datasets and code, this study ensures reproducibility and practical applicability for the broader research community. Our approach allows us to leverage foundational architectures, such as VisualBert [47], a relatively simple framework that draws on self-attention mechanisms to discern relationships between text and image, and CLIP, a framework recognized for its contrastive approach to classification tasks in a zero-shot manner, and provides a strong baseline for our continued research. This can include testing novel architectures in the future, such as Flamingo [74] and Bootstrapping Language-Image Pre-Training [75], and evaluating how to incorporate the strengths of varying models to improve overall performance. With this multimodal research approach for gauging social media sentiment and discourse across various social media platforms targeting specific identities, we aim to set foundational building blocks that will serve as the groundwork for this type of study and help provide guidance for other researchers who plan to embark on similar endeavors. With the changing landscape of social media moving toward less moderation and restriction of posts, it is now more important than ever to track and investigate the impact of hateful content on marginalized communities.

Previous research analyzing 55,844,310 publicly available, race-related tweets from 2011 to 2021 found a 16.5% increase in negative sentiment at the national level during this period. Tweets referencing Middle Eastern and Black people had the highest proportion of negative sentiment. Furthermore, changes in negative racial sentiment were aligned with events salient to specific groups. For example, there were increases in negative racial sentiment tweets referencing Latinx people from 2015 to 2018, peaking at the end of 2018 with the midterm elections and national discussions of the border wall and immigration [13]. Spikes in negative sentiment for tweets referencing Asian people were observed in March 2020 with the emergence of the COVID-19 pandemic and the use of stigmatizing language, such as the China virus [76]. Social media posts from New York City from 2019 to 2022 found temporal associations between anti-Asian sentiment and anti-Asian hate crimes [77]. Moreover, another study revealed that residents living in states with higher anti-Black tweets had higher measures of implicit and explicit racial bias (eg, favoring an explanation that racial disparities were due to a lack of will and not systemic discrimination) [78]. With ongoing national discussions related to LGBTQIA+ and racially minoritized groups, it is important to continue to track these trends. Building upon this area will improve the available tools to identify posts that reference marginalized groups to express negative or hateful content as well as positive and antihateful content.

Previous studies have underscored how current social media hate speech moderation has significant limitations. Kwarteng et al [79] revealed that automated hate speech detection tools, such as HateSonar and Perspective API (Google LLC), solely detected 17% of all misogynistic tweets targeting Black women. In addition, the inherent complexity and variation of digital hate speech further constrain the detection efficacy of automated social media moderation [80]. Thus, it is imperative to leverage novel ML and natural language processing models to facilitate automated hate speech detection on social media. Improving the efficacy, accuracy, and precision of these models may propel future integration into social media platforms to detect digital hate speech in real time and mitigate its impact on marginalized groups. This study introduces potential models that can be further optimized to improve social media hate speech moderation.

Given the sensitive content of hate speech, it is essential to consider ethical concerns, such as bias, fairness, and privacy. This study used a diverse array of race and LGBTQIA+ hate speech keywords to help ensure a representative sample. In addition, the training posts were independently labeled by trained annotators, and any conflicting annotations were resolved after comprehensive group discussions. These measures were used to minimize the potential introduction of bias into the supervised ML models. As algorithmic biases remain prevalent concerns with novel AI models, implementing ethical safeguards may uphold individual privacy and minimize bias in social media hate speech detection models.

However, the study has certain limitations that warrant discussion. While the reliance on data from a variety of platforms, such as Twitter, Facebook, and Instagram, may be extensive, it excludes other social media platforms. Moreover, the dataset primarily consists of English-language social media posts, which may limit its applicability to multilingual or non-English contexts where linguistic and cultural nuances could significantly influence sentiment and hate detection. In addition, CrowdTangle and Twitter limit data collection to publicly available posts and exclude users who make their posts private. Considering heightened risks associated with data privacy, which are acutely felt by minority communities [81,82], and the discrimination and harassment they endure [83,84], this could incline some individuals toward private accounts, potentially leading to biased data that may not fairly reflect discourses and sentiments expressed by such communities.

We used keyword filtering to identify discourse pertaining to or targeting a specific race or ethnic group, gender identity, or sexual orientation. Although our keyword list is comprehensive, it is not an all-encompassing list. This is particularly relevant in the realm of social media platforms, which provide a landscape where language evolves rapidly. Furthermore, paralinguistic communication, such as emojis, is excluded from the text during the cleaning process, which can lead to further omission of subtle but potentially relevant information.

Despite the efforts of standardizing the annotation process through a detailed codebook and the use of multiple annotators to reach consensus while labeling each post, it is important to note that the classification of sentiment, hate, and antihateful content is subjective and can be influenced by the experiences and biases of annotators. Data augmentation techniques, such as LDA and SMOTE, enhanced model performance. SMOTE particularly helps with class imbalance by generating synthetic examples; however, these examples may not address underlying biases in the training data or reflect realistic hate speech data. These difficulties likely stem from the inherent approaches of the techniques and the complexity of detecting subtle interactions between text and image components. In addition, models may not capture intricate nuances and accurate sentiments, such as sarcasm and satire. The computational resources required for training multimodal models pose potential barriers for researchers and institutions with limited access to high-performance facilities.

Future research can build upon these findings by refining fusion strategies and leveraging advanced transfer learning techniques to improve model adaptability and robustness across diverse datasets and platforms. Furthermore, it is essential to examine performance variations across different marginalized groups, such as racial minority groups, gender identities, and sexual orientations, to identify potential biases in model predictions and ensure equitable performance across demographic categories. In addition, expanding datasets to include multilingual contexts and additional modalities, such as audio and video, would enhance the models’ ability to capture the richness of multimodal social media content. While we did not conduct a dedicated interpretability analysis in this work, models such as CLIP and VisualBERT offer several established techniques that can be used to gain insights into their decision-making processes. For example, attention visualization, gradient-based attribution, and probing of intermediate representations have been commonly used to interpret these models. Dang et al [85] and Madasu et al [86] discussed many techniques to analyze the interpretability of multimodal models. Popular techniques used in attention map generation in recent years are gradient-weighted class activation mapping [87], score-weighted class activation mapping [88], and SmoothGrad [89]. Methods such as Shapley Additive Explanations [90] and Local Interpretable Model-Agnostic Explanations [91] can also be informative for explaining the output of the ML models using quantitative measures, indicating the distribution of each feature from the input data that contributes to the model decision. These efforts will enrich our understanding of the internal dynamics of the multimodal models and increase confidence in the model prediction results.

### Conclusions

AI models are meant to be fair and neutral, but when the dataset is predominantly concentrated on English-language posts, the model becomes biased. This can result in inequitable content moderation, where non-English posts are either overlooked or excessively flagged due to a lack of contextual understanding. To build a more inclusive and effective dataset, it is essential to expand the dataset by including culturally diverse data. One approach is to curate a dataset with multiple languages, regional dialects, and a mixture of 2 languages (eg, Hinglish), allowing models to better capture real-world communication. Beyond linguistic diversity, ensuring accurate labeling is just as important. Native speakers and local experts can provide nuanced annotations that reflect regional expressions and dialectical differences, improving the model’s ability to detect hate speech accurately while minimizing biases in moderation. Expanding data sources beyond Western platforms is a crucial step in addressing these biases. Many AI models rely on English-centric datasets [92] sourced from platforms such as Twitter, Facebook, and Reddit, leading to an overrepresentation of Western discourse patterns. A more balanced approach would include content from non-Western social media platforms, such as Weibo (China), KakaoTalk or Naver Café (South Korea), and VKontakte (Russia), as well as local news sites and regional forums. These sources provide unique linguistic structures, idiomatic expressions, and cultural references that English-centric models often fail to capture [93]. By integrating data from a variety of platforms, AI systems can better understand multilingual discourse, reducing disparities in content moderation and improving fairness across diverse user bases. By addressing these directions, future work can advance the development of inclusive and impactful solutions, advancing hate speech detection and fostering positive web-based interactions.

## Abbreviations

AI: artificial intelligence
API: application programming interface
BERT: Bidirectional Encoder Representations from Transformer
CLIP: Contrastive Language-Image Pretraining
LDA: latent Dirichlet allocation
LGBTQIA+: lesbian, gay, bisexual, transgender, queer, intersex, and asexual
ML: machine learning
ResNet: Residual Network
SMOTE: synthetic minority oversampling technique
VGG-16: Visual Geometry Group 16
VisualBERT: Visual Bidirectional Encoder Representations from Transformer

## Data Availability

The datasets generated or analyzed during this study are not publicly available due to the discontinuation of the tools used to collect them. Twitter data were collected using Twitter’s application programming interface for academic research (now discontinued). More information on applying for access can be found here [39]. Publicly available Facebook and Instagram data were collected using CrowdTangle’s user interface dashboard, a public insights tool from Meta, which is now discontinued. More information can be found here [40].

## References

[1] Ortiz-Ospina E, Roser M The rise of social media. Our World in Data.

[2] Abbas J, Aman J, Nurunnabi M, Bano S (2019). The impact of social media on learning behavior for sustainable education: evidence of students from selected universities in Pakistan. Sustainability.

[3] Pianese T, Belfiore P (2021). Exploring the social networks' use in the health-care industry: a multi-level analysis. Int J Environ Res Public Health.

[4] Gilardi F, Gessler T, Kubli M, Müller S (2021). Social media and political agenda setting. Polit Commun.

[5] Khan W, Ghazanfar MA, Azam MA, Karami A, Alyoubi KH, Alfakeeh AS (2020). Stock market prediction using machine learning classifiers and social media, news. J Ambient Intell Human Comput.

[6] Adarbah H, Al Badi B, Golzar J (2023). The impact of emerging data sources and social media on decision making: a culturally responsive framework. Int J Soc Cult Lang.

[7] Shu K, Sliva A, Wang S, Tang J, Liu H (2017). Fake news detection on social media. SIGKDD Explor Newsl.

[8] McKitrick MK, Schuurman N, Crooks VA (2022). Collecting, analyzing, and visualizing location-based social media data: review of methods in GIS-social media analysis. GeoJournal.

[9] Hohenstein J, Kizilcec RF, DiFranzo D, Aghajari Z, Mieczkowski H, Levy K, Naaman M, Hancock J, Jung MF (2023). Artificial intelligence in communication impacts language and social relationships. Sci Rep.

[10] Klašnja M, Barberá P, Beauchamp N, Nagler J, Tucker JA, Atkeson LR, Alvarez RM (2018). Measuring public opinion with social media data. The Oxford Handbook of Polling and Polling Methods.

[11] Liu C, Tian Y, Shi Y, Huang Z, Shao Y (2024). An analysis of public topics and sentiments based on social media during the COVID-19 Omicron Variant outbreak in Shanghai 2022. Comput Urban Sci.

[12] Kapoor KK, Tamilmani K, Rana NP, Patil P, Dwivedi YK, Nerur S (2017). Advances in social media research: past, present and future. Inf Syst Front.

[13] Nguyen TT, Merchant JS, Yue X, Mane H, Wei H, Huang D, Gowda KN, Makres K, Najib C, Nghiem HT, Li D, Drew LB, Hswen Y, Criss S, Allen AM, Nguyen QC (2024). A decade of tweets: visualizing racial sentiments towards minoritized groups in the United States between 2011 and 2021. Epidemiology.

[14] Christopherson KM (2007). The positive and negative implications of anonymity in internet social interactions: “on the internet, nobody knows you’re a dog”. Comput Human Behav.

[15] Nguyen QC, McCullough M, Meng HW, Paul D, Li D, Kath S, Loomis G, Nsoesie EO, Wen M, Smith KR, Li F (2017). Geotagged US tweets as predictors of county-level health outcomes, 2015-2016. Am J Public Health.

[16] Nguyen TT, Adams N, Huang D, Glymour MM, Allen AM, Nguyen QC (2020). The association between state-level racial attitudes assessed from twitter data and adverse birth outcomes: observational study. JMIR Public Health Surveill.

[17] Nguyen QC, Li D, Meng H, Kath S, Nsoesie E, Li F, Wen M (2016). Building a national neighborhood dataset from geotagged twitter data for indicators of happiness, diet, and physical activity. JMIR Public Health Surveill.

[18] Yeung AW, Kletecka-Pulker M, Eibensteiner F, Plunger P, Völkl-Kernstock S, Willschke H, Atanasov AG (2021). Implications of twitter in health-related research: a landscape analysis of the scientific literature. Front Public Health.

[19] Purba AK, Pearce A, Henderson M, McKee M, Katikireddi S (2024). Social media as a determinant of health. Eur J Public Health.

[20] Yuvaraj N, Srihari K, Dhiman G, Somasundaram K, Sharma A, Rajeskannan S, Soni M, Gaba GS, AlZain MA, Masud M (2021). Nature-inspired-based approach for automated cyberbullying classification on multimedia social networking. Math Probl Eng.

[21] Gandhi A, Adhvaryu K, Poria S, Cambria E, Hussain A (2023). Multimodal sentiment analysis: a systematic review of history, datasets, multimodal fusion methods, applications, challenges and future directions. Inf Fusion.

[22] Farkas X, Bene M (2020). Images, Politicians, and Social Media: Patterns and Effects of Politicians’ Image-Based Political Communication Strategies on Social Media. Int J Press Politics.

[23] DeCook JR (2018). Memes and symbolic violence: #proudboys and the use of memes for propaganda and the construction of collective identity. Learn Media Technol.

[24] Huntington HE (2013). Subversive memes: internet memes as a form of visual rhetoric. AoIR Selected Papers of Internet Research.

[25] Dawkins R (2016). The Selfish Gene. 4th edition.

[26] Das A, Wahi JS, Li S Detecting hate speech in multi-modal memes. arXiv.

[27] Jennifer C, Tahmasbi F, Blackburn J, Stringhini G, Zannettou S, Cristofaro E Feels bad man: dissecting automated hateful meme detection through the lens of Facebook's challenge. arXiv.

[28] Habash M, Daqour Y, Abdullah M, Al-Ayyoub M (2022). YMAI at SemEval-2022 task 5: detecting misogyny in memes using VisualBERT and MMBT MultiModal pre-trained models. Proceedings of the 16th International Workshop on Semantic Evaluation.

[29] Kiela D, Firooz H, Mohan A The hateful memes challenge: detecting hate speech in multimodal memes. arXiv.

[30] Kumar GK, Nandakumar K Hate-CLIPper: multimodal hateful meme classification based on cross-modal interaction of CLIP features. arXiv.

[31] Wu F, Chen G, Cao J, Yan Y, Li Z (2024). Multimodal hateful meme classification based on transfer learning and a cross-mask mechanism. Electronics.

[32] Burbi G, Baldrati A, Agnolucci L, Bertini M, Bimbo A Mapping memes to words for multimodal hateful meme classification. arXiv.

[33] Kirk H, Jun Y, Rauba P, Wachtel G, Li R, Bai X, Broestl N, Doff-Sotta M, Shtedritski A, Asano Y (2021). Memes in the wild: assessing the generalizability of the hateful memes challenge dataset. Proceedings of the 5th Workshop on Online Abuse and Harms.

[34] Sharma S, Alam F, Akhtar M, Dimitrov D, Martino G, Firooz H, Halevy A, Silvestri F, Nakov P, Chakraborty T Detecting and understanding harmful memes: a survey. arXiv.

[35] Hee MS, Chong WH, Lee RK (2023). Decoding the underlying meaning of multimodal hateful memes. Proceedings of the 32nd International Joint Conference on Artificial Intelligence.

[36] Hee MS, Lee RK, Chong WH (2022). On explaining multimodal hateful meme detection models. Proceedings of the Conference 2022 on ACM Web.

[37] Hossain E, Sharif O, Hoque M, Preum S (2024). Deciphering hate: identifying hateful memes and their targets. Proceedings of the 62nd Annual Meeting of the Association for Computational Linguistics.

[38] Yang SH, Chen CC, Huang HH, Chen HH Entity-aware dual co-attention network for fake news detection. arXiv.

[39] Starting February 9, we will no longer support free access to the Twitter API, both v2 and v1.1. A paid basic tier will be available instead. X Developers.

[40] Exclusive: Elon Musk's X restructuring curtails disinformation research, spurs legal fears. Reuters.

[41] What is Amazon Rekognition?. Amazon Rekognition.

[42] JaidedAI / EasyOCR. GitHub.

[43] Open source data labeling. Label Studio.

[44] Liang T, Lin G, Wan M, Li T, Ma G, Lv F Expanding large pre-trained unimodal models with multimodal information injection for image-text multimodal classification. CVRP.

[45] Devlin D, Chang MW, Lee K, Toutanova K BERT: pre-training of deep bidirectional transformers for language understanding. arXiv.

[46] Tammina S (2019). Transfer learning using VGG-16 with deep convolutional neural network for classifying images. Int J Sci Res.

[47] Li LH, Yatskar M, Yin D, Hsieh CJ, Chang KW VisualBERT: a simple and performant baseline for vision and language. arXiv.

[48] News. Common Objects in Context.

[49] Agrawal A, Lu J, Antol S, Mitchell M, Zitnick CL, Batra D, Parikh D VQA: visual question answering. arXiv.

[50] Boulahia SY, Amamra A, Madi MR, Daikh S (2021). Early, intermediate and late fusion strategies for robust deep learning-based multimodal action recognition. Mach Vis Appl.

[51] Radford A, Kim JW, Hallacy C, Ramesh A, Goh G, Agarwal S, Sastry G, Askell A, Mishkin P, Clark J, Krueger G, Sutskever I Learning transferable visual models from natural language supervision. arXiv.

[52] Otten NV What is FastText? Compared to word2vec and glove [how to tutorial in Python]. Spot Intelligence.

[53] UMDPublicHealth/Twitter-Tweets-Collection-And-PostProcessing: Twitter tweets collection and post-processing, including keyword filtering, variables cleaning up, sentiment testing etc. GitHub.

[54] AdamW — PyTorch 2.6 documentation. PyTorch.

[55] Khan AA, Chaudhari O, Chandra R (2024). A review of ensemble learning and data augmentation models for class imbalanced problems: combination, implementation and evaluation. Expert Syst Appl.

[56] ColorJitter — torchvision main documentation. PyTorch.

[57] RandomHorizontalFlip — torchvision main documentation. PyTorch.

[58] RandomRotation — torchvision main documentation. PyTorch.

[59] Dablain D, Jacobson KN, Bellinger C, Roberts M, Chawla NV (2023). Understanding CNN fragility when learning with imbalanced data. Mach Learn.

[60] He K, Zhang X, Ren S, Sun J Deep residual learning for image recognition. arXiv.

[61] Residual neural network - an overview. ScienceDirect.

[62] AdaptiveAvgPool2d — PyTorch 2.6 documentation. PyTorch.

[63] Efraimidis P, Spirakis P, Kao MY (2008). Weighted random sampling. Encyclopedia of Algorithms.

[64] Mannor S, Peleg D, Rubinstein R (2005). The cross entropy method for classification. Proceedings of the 22nd international conference on Machine learning.

[65] Lin TY, Goyal P, Girshick R, He K, Dollár P Focal loss for dense object detection. arXiv.

[66] Chawla NV, Bowyer KW, Hall LO, Kegelmeyer WP (2002). SMOTE: synthetic minority over-sampling technique. J Artif Intell Res.

[67] Blei DM, Ng AY, Jordan MI (2003). Latent Dirichlet allocation. J Mach Learn Res.

[68] Liu P, Wang X, Xiang C, Meng W (2020). A survey of text data augmentation. Proceedings of the 2020 International Conference on Computer Communication and Network Security.

[69] Grandini M, Bagli E, Visani G Metrics for multi-class classification: an overview. arXiv.

[70] Cao R, Lee RK, Chong WH, Jiang J Prompting for multimodal hateful meme classification. arXiv.

[71] HarMeme dataset. Papers With Code.

[72] Gottfried J Americans’ social media use. Pew Research Center.

[73] Chen Y, Pan F (2022). Multimodal detection of hateful memes by applying a vision-language pre-training model. PLoS One.

[74] Alayrac JB, Donahue J, Luc P, Miech A, Barr I, Hasson Y, Lenc K, Mensch A, Millican K, Reynolds M, Ring R, Rutherford E, Cabi S, Han T, Gong Z, Samangooei S, Monteiro M, Menick J, Borgeaud S, Brock A, Nematzadeh A, Sharifzadeh S, Binkowski M, Barreira R, Vinyals O, Zisserman A, Simonyan K Flamingo: a visual language model for few-shot learning. arXiv.

[75] Li J, Li D, Savarese S, Hoi S BLIP-2: bootstrapping language-image pre-training with frozen image encoders and large language models. arXiv.

[76] Nguyen TT, Criss S, Dwivedi P, Huang D, Keralis J, Hsu E, Phan L, Nguyen LH, Yardi I, Glymour MM, Allen AM, Chae DH, Gee GC, Nguyen QC (2020). Exploring U.S. shifts in anti-Asian sentiment with the emergence of COVID-19. Int J Environ Res Public Health.

[77] Wei H, Hswen Y, Merchant JS, Drew LB, Nguyen QC, Yue X, Mane H, Nguyen TT (2024). From tweets to streets: observational study on the association between twitter sentiment and anti-Asian hate crimes in New York City from 2019 to 2022. J Med Internet Res.

[78] Nguyen TT, Huang D, Michaels EK, Glymour MM, Allen AM, Nguyen QC (2021). Evaluating associations between area-level Twitter-expressed negative racial sentiment, hate crimes, and residents' racial prejudice in the United States. SSM Popul Health.

[79] Kwarteng J, Perfumi SC, Farrell T, Third A, Fernandez M (2022). Misogynoir: challenges in detecting intersectional hate. Soc Netw Anal Min.

[80] Díaz Á, Hecht-Felella L Double standards in social media content moderation. Brennan Center for Justice.

[81] Fiesler C, Dye M, Feuston J, Hiruncharoenvate C, Hutto C, Morrison S, Khanipour RP, Pavalanathan U, Bruckman A, De CM, Gilbert E (2017). What (or who) is public?: privacy settings and social media content sharing. Proceedings of the 2017 ACM Conference on Computer Supported Cooperative Work and Social Computing.

[82] Lai S, Tanner B Examining the intersection of data privacy and civil rights. Brookings.

[83] Scott JE, Barlett CP (2023). Understanding cyber-racism perpetration within the broader context of cyberbullying theory: a theoretical integration. Children (Basel).

[84] Vogels EA The state of online harassment. Pew Research Center.

[85] Dang Y, Huang K, Huo J, Yan Y, Huang S, Liu D, Gao M, Zhang J, Qian C, Wang K, Liu Y, Shao J, Xiong H, Hu X Explainable and interpretable multimodal large language models: a comprehensive survey. arXiv.

[86] Madasu A, Gandelsman Y, Lai V, Howard P Quantifying and enabling the interpretability of CLIP-like models. arXiv.

[87] Selvaraju RR, Cogswell M, Das A, Vedantam R, Parikh D, Batra D (2019). Grad-CAM: visual explanations from deep networks via gradient-based localization. Int J Comput Vis.

[88] Wang H, Wang Z, Du M, Yang F, Zhang Z, Ding S, Mardziel P, Hu X Score-CAM: score-weighted visual explanations for convolutional neural networks. Computer Vision Foundation.

[89] Smilkov D, Thorat N, Kim B, Viégas F, Wattenberg M SmoothGrad: removing noise by adding noise. arXiv.

[90] Lundberg S, Lee SI A unified approach to interpreting model predictions. arXiv.

[91] Ribeiro MT, Singh S, Guestrin C "Why should I trust you?": explaining the predictions of any classifier. arXiv.

[92] Blasi D, Anastasopoulos A, Neubig G Systematic inequalities in language technology performance across the world's languages. arXiv.

[93] Joshi P, Santy S, Budhiraja A, Bali K, Choudhury M (2020). The state and fate of linguistic diversity and inclusion in the NLP world. Proceedings of the 58th Annual Meeting of the Association for Computational Linguistics.

